# Metformin reduces androgen receptor and upregulates homeobox A10 expression in uterine endometrium in women with polycystic ovary syndrome

**DOI:** 10.1186/s12958-021-00765-6

**Published:** 2021-05-31

**Authors:** Miki Ohara, Hiromi Yoshida-Komiya, Miho Ono-Okutsu, Akiko Yamaguchi-Ito, Toshifumi Takahashi, Keiya Fujimori

**Affiliations:** 1 Department of Obstetrics and Gynecology, Fukushima Medical University, School of Medicine, 1 Hikarigaoka, Fukushima, 960-1295 Japan; 2 Center for Gender Specific Medicine, Fukushima Medical University, 1 Hikarigaoka, Fukushima, 960-1295 Japan; 3 Fukushima Medical Center for Children and Women, Fukushima Medical University, 1 Hikarigaoka, Fukushima, 960-1295 Japan

**Keywords:** Polycystic ovary syndrome, Metformin, Uterine endometrium, Androgen receptor, Homeobox A10

## Abstract

**Background:**

Polycystic ovary syndrome (PCOS) causes anovulation and is associated with a reduced clinical pregnancy rate. Metformin, which is widely used for treating PCOS, can lead to successful pregnancy by restoring the ovulation cycle and possibly improving endometrial abnormality during the implantation period. However, the mechanism by which metformin improves endometrial abnormality remains unknown. Women with PCOS have an aberrant expression of steroid hormone receptors and homeobox A10 (HOXA10), which is essential for embryo implantation in the endometrium.

**Methods:**

In this study, we examined whether metformin affects androgen receptor (AR) and HOXA10 expression in PCOS endometrium in vivo and in human endometrial cell lines in vitro. Expression of AR and HOXA10 was evaluated by immunohistochemistry, fluorescent immunocytochemistry, and western blot analysis.

**Results:**

AR expression was localized in both epithelial and stromal cells; however, HOXA10 expression was limited to only stromal cells in this study. In women with PCOS, 3 months after metformin treatment, the expression of AR was reduced in epithelial and stromal cells in comparison to their levels before treatment. In contrast, HOXA10 expression in the stromal cells with metformin treatment increased in comparison to its level before treatment. Further, we showed that metformin counteracted the testosterone-induced AR expression in both Ishikawa cells and human endometrial stromal cells (HESCs); whereas, metformin partly restored the testosterone-reduced HOXA10 expression in HESCs in vitro.

**Conclusions:**

Our results suggest that metformin may have a direct effect on the abnormal endometrial environment of androgen excess in women with PCOS.

**Trial registration:**

The study was approved by the Ethical Committee of Fukushima Medical University (approval no. 504, approval date. July 6, 2006), and written informed consent was obtained from all patients. https://www.fmu.ac.jp/univ/sangaku/rinri.html

## Background

Polycystic ovary syndrome (PCOS) is a common disorder that affects 10% of women of reproductive age. Women with PCOS have infertility due to anovulation and metabolic disorders, such as dyslipidemia, glucose intolerance, and metabolic syndrome [1]. Even if anovulation is restored in women with PCOS, live birth rate is not high because of a high miscarriage rate of 30–50% [2, 3].

Women with PCOS have uterine endometrial abnormalities such as reduced pregnancy rate, endometrial hyperplasia, and endometrial cancer due to an abnormal steroid hormone environment [1–4]. Women with PCOS show unopposed hyperestrogenemia caused by anovulation, and hyperandrogenemia [1, 2]. Abnormal spatiotemporal expression of steroid hormone receptors, such as estrogen receptor, progesterone receptor, androgen receptor (AR), and steroid receptor cofactors in the endometrium have been reported in women with PCOS [2, 4–6]. However, there are only few studies on how pharmacotherapy affects an abnormal steroid hormone environment of the endometrium in women with PCOS.

Metformin, a biguanide insulin-sensitizing drug, is used worldwide in women with PCOS. Metformin treatment results in successful pregnancy by restoring the ovulation cycle and possibly improving endometrial abnormalities. Ovulatory cycle is restored by reducing peripheral insulin resistance by metformin [7–10]. However, it remains unknown how metformin improves endometrial abnormality [10, 11]. Although we have previously shown that metformin treatment decreases the level of AR expression in the endometrium of women with PCOS [10], the direct effect of metformin on the endometrium is still unclear.

Homeobox A10 (HOXA10), a member of the homeobox superfamily of transcription factors, is indispensable for the implantation of embryos by differentiation of the endometrium [12, 13]. HOXA10 expression in the endometrium is regulated by steroid hormones. Ovarian steroid hormones, such as estrogen and progesterone induce HOXA10 expression in women with normal menstrual cycles [12–15]. Testosterone inhibits HOXA10 expression in the endometrium of women with PCOS [16]. However, there are very few reports that have examined the direct effect of HOXA10 expression and testosterone on the endometrium in women with PCOS.

In this study, we aimed to determine whether metformin affects AR and HOXA10 expression in the endometrium of women with PCOS in vivo and human endometrial cell lines in vitro.

## Methods

### Patient recruitment and tissue collection

Diagnosis of PCOS was based on the Rotterdam criteria, wherein, the presence of two of the three following characteristics is required for inclusion: (1) oligomenorrhea/amenorrhea, (2) chemical or clinical findings of hyperandrogenism, and (3) polycystic ovaries observed on transvaginal sonography [17]. Patients who had received oral contraceptives or other drugs up to 3 months before entering the study were excluded. Endometrial tissue samples were collected via suction aspiration biopsy both before and after metformin treatment in three women (cases 1, 2, and 3) with PCOS. All three patients manifested oligomenorrhea/ amenorrhea and presented polycystic ovaries; however, chemical/ clinical findings of hyperandrogenism were observed only in case 2. We administered metformin at 750 mg/day for 3 months to patients with PCOS, and sampled their endometrium and measured their endocrine/glucose metabolism parameters both before and after metformin treatment [10]. For non-PCOS patients, we collected five samples of endometrial tissues from patients (two in the proliferative phase and three in the secretory phase) of reproductive age undergoing hysterectomy for uterine fibroids and/or adenomyosis.

This study was approved by the Ethical Committee of Fukushima Medical University (approval No. 504). All patients provided written informed consent for publication of their data in this paper.

### Immunohistochemistry

Specimens embedded in paraffin were sliced to 5 μM. Immunostaining was performed using the procedure described in our previous study [10]. Briefly, the tissue sections were deparaffinized with xylene, 10 mM citrate buffer (pH 6.0) was heated to activate the antigens, and endogenous peroxidase was removed using 3% hydrogen peroxide-ethanol for 5 min. After blocking for 60 min, the primary antibody was incubated overnight at 4 °C. AR (rabbit polyclonal antibody, #Sc-815, Santa Cruz Biotechnology, Dallas, TX, USA) and HOXA10 (goat polyclonal antibody, #Sc-17,159, Santa Cruz Biotechnology) antibodies were used as primary antibodies. For negative controls, no primary antibodies were used. Secondary antibodies (anti-rabbit antibody for AR and anti-goat antibody for HOXA10) were allowed to react at room temperature (20–25 °C) for 60 min. The blocking solutions and secondary antibodies were used from the VECTASTAIN *Elite* ABC IgG Kit (AR: #PK-6101, HOXA10: #PK-6105, Vector Laboratories, Burlingame, CA, USA). The avidin-biotinylated peroxidase complex so formed was incubated for 20 min. A color reaction was carried out using a 3,3′-diaminobentidine (#D5905, Sigma-Aldrich, St. Louis, MO, USA) solution, and nuclear staining was performed using hematoxylin.

The slides were observed under an upright microscope (Olympus BX51, Olympus, Tokyo, Japan), and endometrial classification was performed using the Noyes criteria [18]. The staining intensity was evaluated using a histochemical scoring system (HSCORE) [19] and semi-quantified. The staining density was categorized into five stages as follows: 0 (zero), 1+ (weak), 2+ (moderate), 3+ (strong), and 4+ (very strong). Distribution of each positive cell was expressed in percentage (P0, P1, P2, P3, and P4), and HSCORE was calculated as: HSCORE = Σ Pi (i + 1), where i = 0, 1, 2, 3, 4 (Pi = 0–100%). Calculation using this formula was performed using an independent intra-observer (AR: *r* = 0.96, HOXA10: *r* = 0.88), inter observer (AR: *r* = 0.94, HOXA10: *r* = 0.92). HSCORE values before and after metformin treatment were compared.

### Cell culture and experimental treatments

Human endometrial epithelial cell line, Ishikawa cells (kindly provided by Kasumigaura Medical Center, Ibaraki, Japan), and human endometrial stromal cells (HESCs; ATCC^R^CRL-4003™, American Type Culture Collection, Manassas, VA, USA) were used as the epithelial cell model and stromal cell model, respectively. Both cell lines have endometrial characteristics, and have been used in previous studies that involve endometrial functions, such as proliferation, decidualization, and apoptosis of endometrial stroma [20–24]. Ishikawa cells were maintained in Dulbecco’s Modified Eagle’s Medium (DMEM)-low glucose (without phenol red, #D5921, Sigma-Aldrich), and supplemented with 10% fetal bovine serum (FBS, #10099–141, Gibco, Thermo Fisher Scientific, Waltham, MA, USA), 2% L-glutamine (#G7513, Sigma-Aldrich), and 1% penicillin-streptomycin mixture (#168–23191, Wako, Osaka, Japan). HESCs were maintained in DMEM/nutrient mixture F-12 Ham (without phenol red, #D2906, Sigma-Aldrich), and supplemented with 10% FBS, 0.15% sodium bicarbonate (#S5761, Sigma-Aldrich), 1% ITS + premix (c#354352, Corning Inc., Corning, NY, USA), and 0.005% puromycin (#P9620, Sigma-Aldrich). Both cell lines were incubated in a humidified incubator at 37 °C with 5% CO_2_.

Ishikawa cells and HESCs were seeded at a concentration of 2 × 10^4^ cells/cm^2^. Twenty-four hours after plating, the cells were serum-starved for an additional 24 h. Then, testosterone (#T1500, Sigma-Aldrich) and metformin (#1115-70-4, Wako, Osaka, Japan) were added. After 48 h of treatment, the cells were harvested and used for subsequent experiments. Testosterone was dissolved in ethanol, diluted in phosphate-buffered saline (PBS) to a final concentration of 1.0 mM, and stored at − 20 °C until further use. Testosterone concentration used in the experiment was 0.01 mM. In preliminary experiments, the concentration of ethanol, which was used as a vehicle, was equivalent to 0.58%; this concentration of ethanol did not affect the cell culture or the experimental results. Metformin was dissolved in PBS (−), and 1.0 mM, 10^− 1^ mM, and 10^− 2^ mM concentrations were used.

### Western blotting

Proteins were extracted using T-PER (#78510, Thermo Fisher Scientific) supplemented with a protease inhibitor cocktail tablet (cOmplete, Mini, EDTA-free, #11836170001, Roche Diagnostics, Mannheim, Germany). Equal quantities of protein were resolved on Mini-PROTEAN TGX precast gels (4–15%, #4561083, Bio-Rad Laboratories Inc., Hercules, CA, USA), subjected to sodium dodecyl sulfate-polyacrylamide gel electrophoresis (100 V, 60 min), and then transferred onto a 0.2 μM polyvinylidene difluoride (PVDF) membrane using a Trans-Blot Turbo Transfer Pack (#1704156, Bio-Rad Laboratories Inc.; 25 V, 10 min). The membranes were washed with 0.1% Tris-buffered saline supplemented with Triton-X (TBS-T; #P1379, Sigma-Aldrich), and treated with 5% nonfat dry milk/TBS-T for 60 min. Primary antibodies for AR (#ab133273, rabbit monoclonal antibody, Abcam, Cambridge, UK) and HOXA10 (#sc-17,159, goat polyclonal antibody, Santa Cruz Biotechnology) were allowed to react overnight at 4 °C. No antibodies were added in the negative controls. After washing, the secondary antibodies (AR: #ab97080, anti-rabbit antibody, Abcam; HOXA10: #sc-2020, anti-goat antibody, Santa Cruz Biotechnology) were incubated for 1 h at room temperature. Chemiluminescence was performed using ECL Prime Western Blotting Detection reagent (#RPN2232, GE Healthcare, Piscataway, NJ, USA), and imaging was performed with Amersham Imager (GE Healthcare). PVDF membranes were stripped using Restore™ PLUS Western Blot Stripping Buffer (#46430, Thermo Fisher Scientific), and re-probed with GAPDH (#ab9485, polyclonal antibody, Abcam). The band intensities were digitized, and AR or HOXA10/GAPDH values were calculated for quantitative evaluation. These experiments were conducted thrice, with similar results.

### Fluorescent immunocytochemistry

After the cells were seeded onto 8-well chamber slides, testosterone and metformin were added. The cells were washed with PBS (−) and fixed in 4% paraformaldehyde/PBS (#163–20,145, Wako) at room temperature for 15 min and permeabilized with PBS/0.5% Triton-X. The primary antibody was diluted with 3% bovine serum albumin (BSA)/PBS (−) and incubated at 4 °C overnight. The secondary antibody (donkey anti-rabbit IgG H&L [Alexa Fluor 488] donkey polyclonal secondary antibody, #ab150073; Abcam) was incubated for 60 min at room temperature. After probing with each antibody, the slides were finally encapsulated with 4′ 6-diamidino-2-phenylindole (DAPI)-added mounting agent, Pro Long Gold Antifade Mountant with DAPI (#P36941, Thermo Fisher Scientific). A confocal laser microscope system (OLYMPUS FV1000, Olympus) was used for imaging.

### Statistical analysis

All statistical analyses were performed using EZR (Saitama Medical Center, Jichi Medical University, Saitama, Japan) [25]. HSCORE values are presented as mean ± standard deviation. Analysis of variance was applied to compare the changes in AR and HOXA10 expression levels between different groups. Differences were considered significant at *P* < 0.05.

## Results

### Patient characteristics in women with PCOS before and after metformin treatment

Age, body mass index, and endocrine and metabolic parameters in three women with PCOS are shown in Table 1, which includes endometrial classification and pregnancy outcomes in our previous study [10]. Level of luteinizing hormone in cases 2 and 3 decreased after metformin treatment compared to that before treatment. Level of free testosterone and homeostasis model assessment-insulin resistance (HOMA-IR) decreased in each case after metformin treatment.

**Table 1:** Patient characteristics in women with PCOS before and after metformin treatment

Metformin treatment	Case 1		Case 2		Case 3	
	Before	After	Before	After	Before	After
**Age (years)**	25		31		32	
**BMI (kg/m**^**2**^**)**	25.8	25.1	27.2	25.0	26.3	27.1
**LH (mIU/ml)**	9.9	10.13	8.19	3.06	7.50	2.51
**FSH (mIU/ml)**	5.12	6.25	4.73	4.48	5.62	5.52
**Free testosterone (pg/ml)**	1.6	1.4	2.4	0.8	1.0	0.6
**DHEA-S (μg/ml)**	128	287	109	129	157	–
**Estradiol (pg/ml)**	45	46	42	33	41	44
**HOMA-IR**	1.97	0.97	2.48	1.6	1.72	1.67
**Endometrial classification**	proliferative phase	proliferative phase	proliferative phase	secretory phase	proliferative phase	proliferative phase
**Pregnancy outcome**		unknown		conception after 8 months from the beginning of metformin treatment		conception after 17 months from the beginning of metformin treatment

PCOS, polycystic ovary syndrome; BMI, body mass index; LH, luteinizing hormone; FSH, follicle stimulating hormone; DHEA-S, dehydroepiandrosterone sulfate; HOMA-IR, homeostasis model assessment of insulin resistance (This cited a part of Table 2 in our previous report [10])

Histological analysis after metformin treatment revealed the presence of an early secretory phase endometrium only in case 2 (data not shown); at the time of endometrial biopsy, an increase in the patient’s basal body temperature was observed and ultrasonography revealed a hyperechoic endometrium corresponding with the secretory phase. Two women with PCOS, who were treated with metformin for more than 3 months, became pregnant and gave birth.

### Changes in immunohistochemical expression patterns of AR and HOXA10 in non-PCOS women

**Fig. 1:**
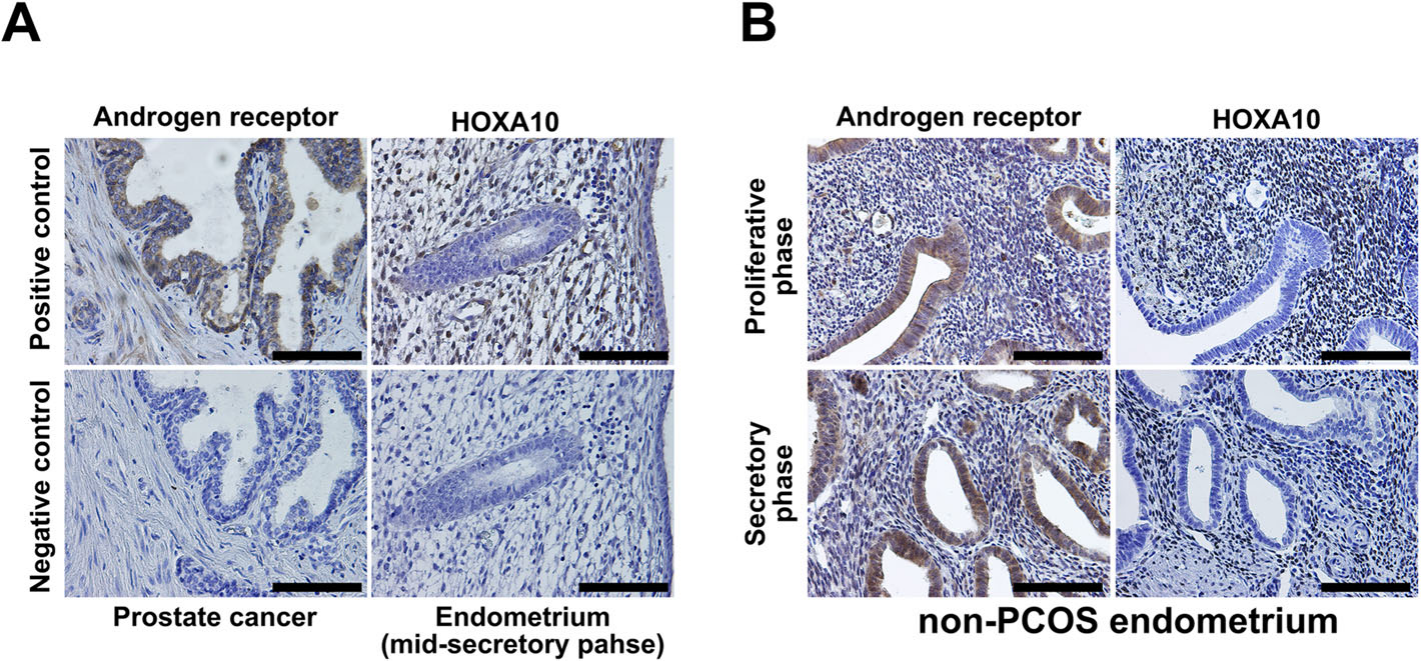
Expression levels of AR and HOXA10 determined through immunohistochemistry in endometrium tissues of non-PCOS women. **a.** Expression of AR and HOXA10 in controls. Images in the upper panel show positive controls and images in the lower panel show negative controls (AR for prostate cancer; HOXA10 for endometrium from mid-secretory phase). **b.** Representative images of AR and HOXA10 expression in the endometrium obtained from non-PCOS women. Images in the upper panel show AR and HOXA10 expression in the proliferative phase, and images in the lower panel show AR and HOXA10 expression in the secretory phase. Scale bar = 100 μM. HOXA10, homeobox A10; PCOS, polycystic ovary syndrome; AR, androgen receptor

As no researchers have reported the expression patterns of AR and HOXA10 in the same endometrial samples acquired from non-PCOS women, we performed immunohistochemical analysis for determining AR and HOXA10 endometrial expression in five non-PCOS women. As shown in Fig. 1b, AR was expressed in the nuclei of both epithelial and stromal cells, and HOXA10 was expressed only in the nuclei of stromal cells. No significant differences were found in AR expression in the epithelial and stromal cells between the proliferative and secretory phases (Fig. 3); whereas, HOXA10 expression in stromal cells in the secretory phase was higher than that in the proliferative phase (Fig. 3).

### Changes in immunohistochemical expression patterns of AR and HOXA10 in women with PCOS before and after metformin treatment

**Fig. 2:**
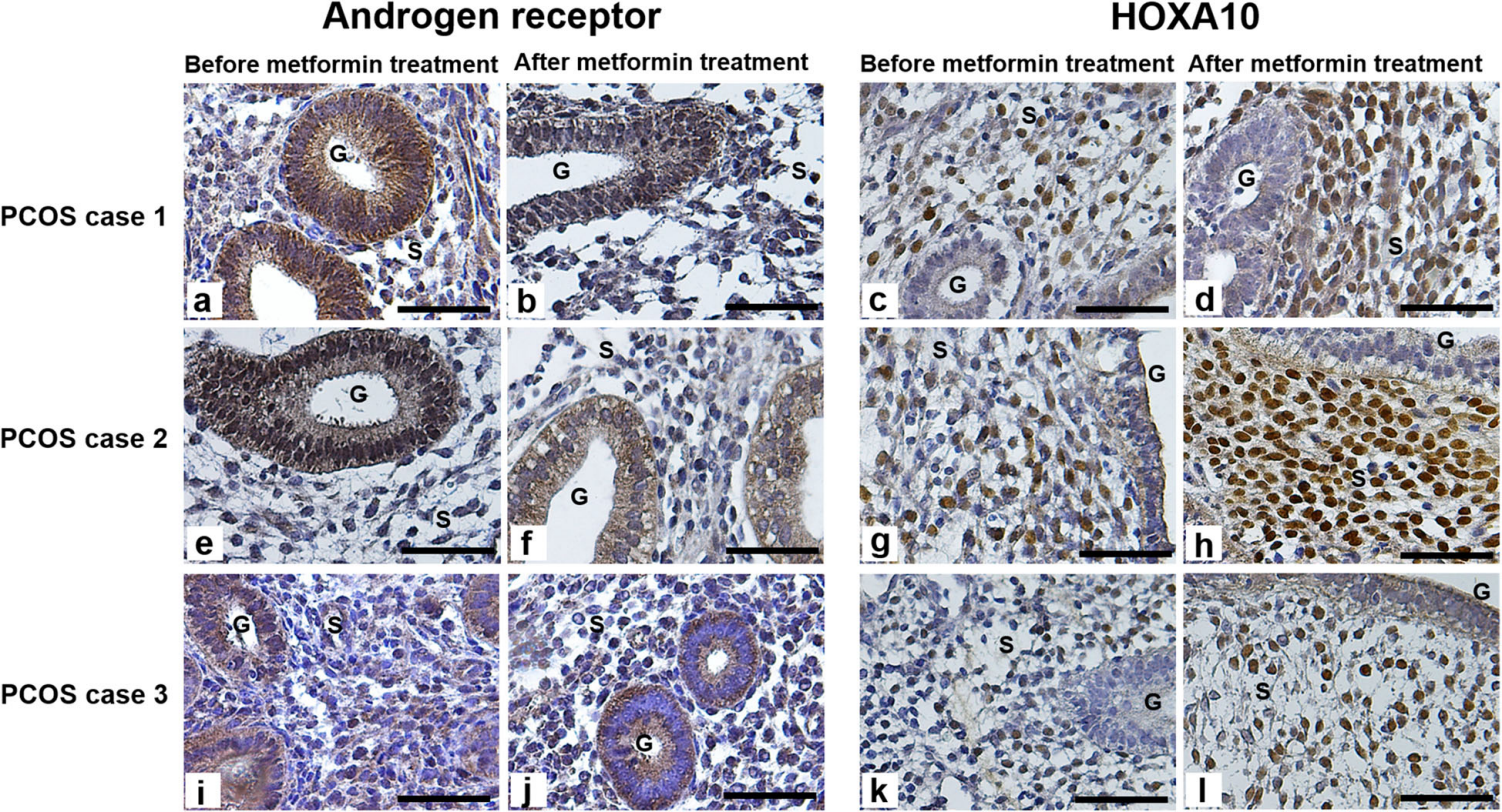
Metformin treatment changes expression of AR and HOXA10 in endometrium tissues from women with PCOS. Representative images of immunohistochemical expression of AR and HOXA10 in the endometrial tissues obtained from three women with PCOS (cases 1, 2, and 3) before and after 3 months of metformin treatment. On the left, the images show AR expression before (**a**, **e**, **i**) and after metformin treatment (**b**, **f**, **j**). On the right, the images show HOXA10 expression before (**c**, **g**, **k**) and after metformin treatment (**d**, **h**, **l**). Scale bar = 50 μM. HOXA10, homeobox A10; PCOS, polycystic ovary syndrome; AR, androgen receptor. G, glandular epithelium; S, stroma

**Fig. 3:**
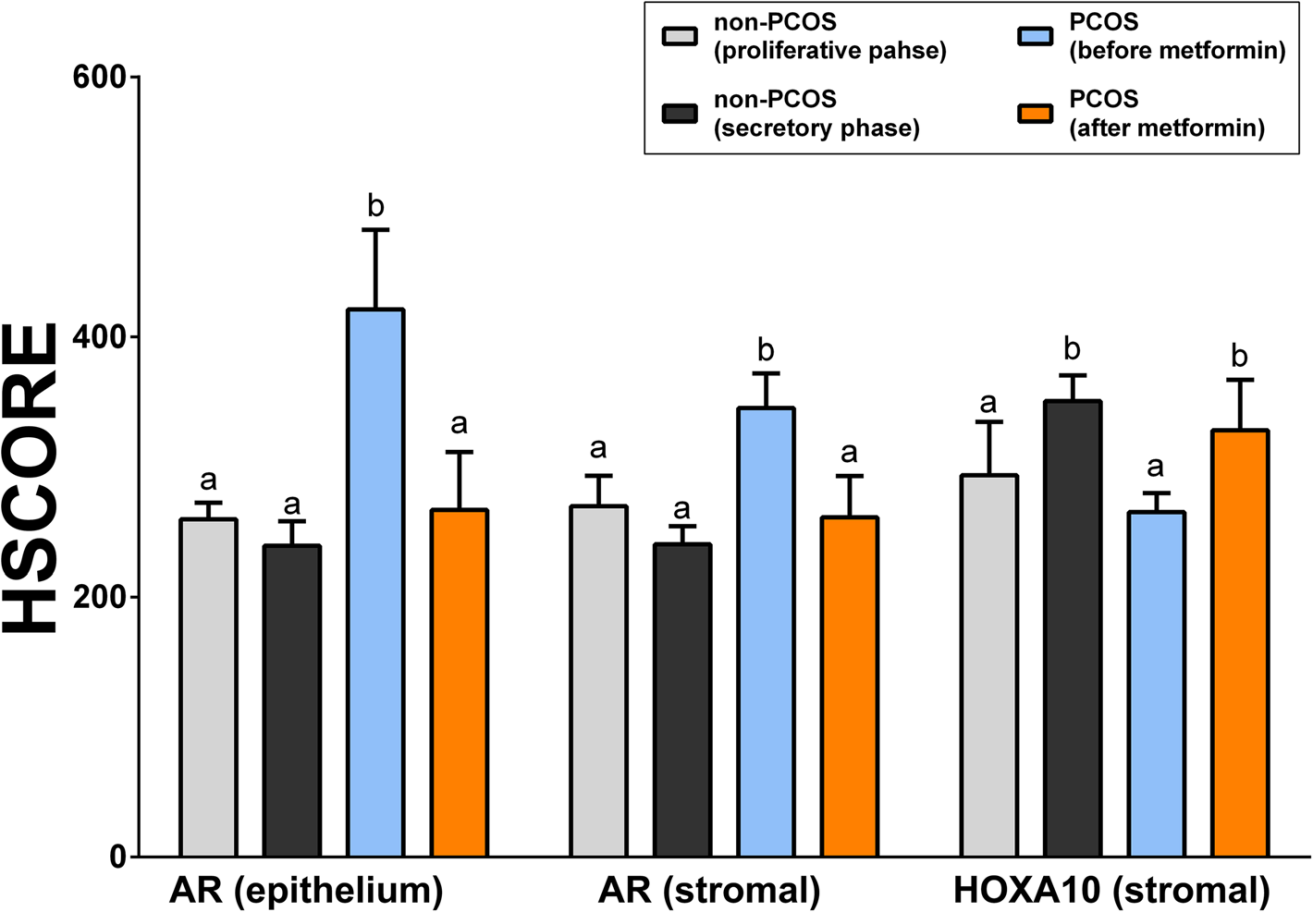
Quantification of immunoexpression of AR and HOXA10 in endometrium tissues from non-PCOS and PCOS women. Graphs show quantification of AR and HOXA10 immunoexpression in the proliferative (*n* = 2) and secretory phase (*n* = 3) endometrium tissues obtained from non-PCOS women and those before and after 3 months of metformin treatment in women with PCOS (*n* = 3). The experiments were performed in triplicate for each sample. Different letters above the bars indicate the significant differences at *P* < 0.05. AR, androgen receptor; HOXA10, homeobox A10; HSCORE, histological score; PCOS, polycystic ovary syndrome

Immunohistochemical analysis was performed to determine AR and HOXA10 expressions in the endometrial cells obtained from women with PCOS (Fig. 2). Levels of AR expression in both epithelial and stromal cells after metformin treatment were significantly (*P* < 0.05) decreased compared with their levels before metformin treatment (Fig. 3). Whereas, the level of HOXA10 expression in the stromal cells after metformin treatment was significantly increased compared with its level before metformin treatment (Fig. 3). These data showed that metformin treatment decreased AR expression and increased HOXA10 expression in PCOS endometrium.

### Metformin counteracts testosterone-induced AR expression in the epithelial and stromal cell lines

**Fig. 4:**
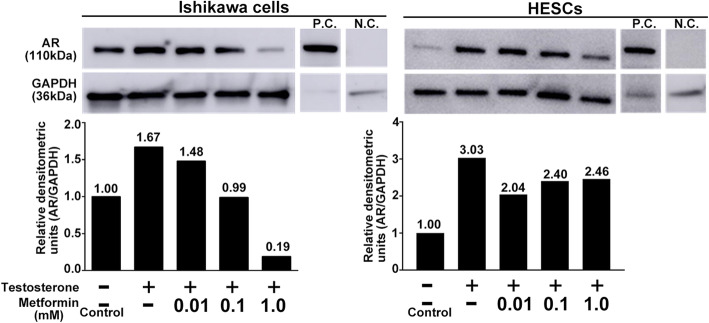
Effect of metformin on AR expression in Ishikawa cells and HESCs examined by western blotting. Representative images show AR expression by western blotting in Ishikawa cells and HESCs in the upper panel. Mouse testicular lysate was used as a positive control, and no primary AR antibody was added in the negative control. Graphs in the lower panel show the relative densitometric units (AR/GAPDH). The value in each column is the relative ratio when the control (without both testosterone and metformin) was set at 1.0. Three independent experiments showed the same results. AR, androgen receptor; HESCs, human endometrial stromal cells; N.C., negative control; P.C., positive control

**Fig. 5:**
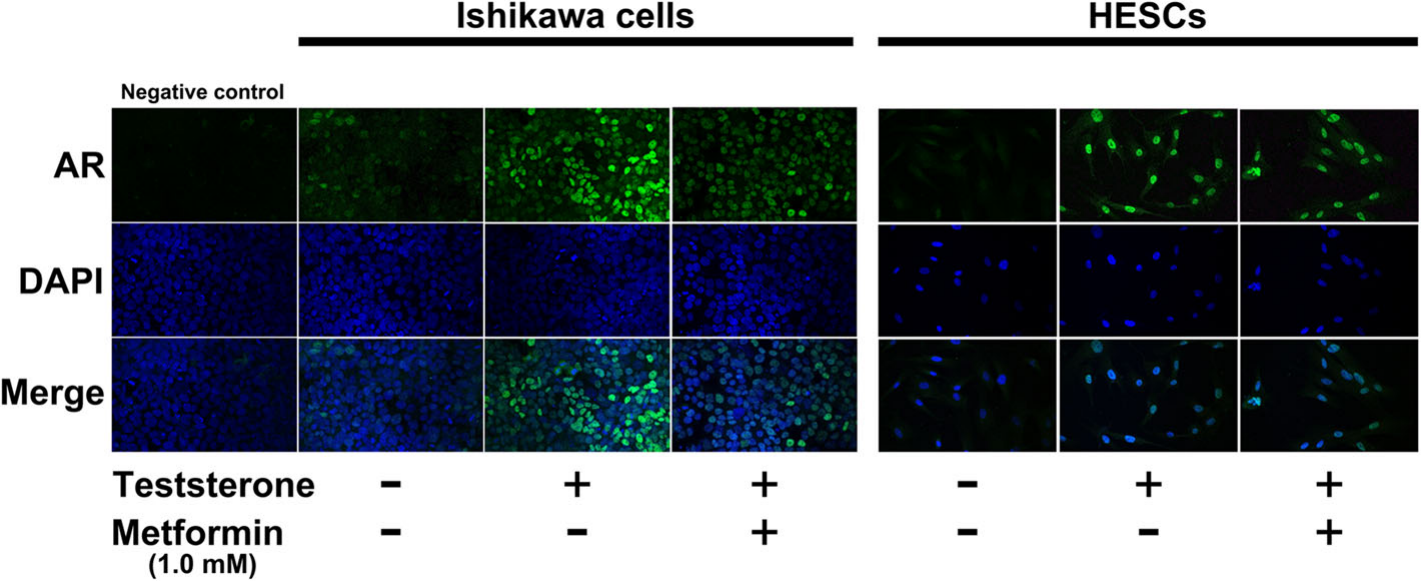
Effect of metformin on AR expression in Ishikawa cells and HESCs examined by fluorescent immunocytochemistry. Representative images of fluorescent immunocytochemistry in Ishikawa cells and HESCs. Samples without the AR primary antibody were used as a negative control. AR, androgen receptor; DAPI, 4′ 6-diamidino-2-phenylindole; HESCs, human endometrial stromal cells

We investigated the direct effects of metformin on AR expression in endometrium in vitro. Ishikawa cells and HESCs exposed to testosterone were used as an endometrial model for women with PCOS. Testosterone treatment resulted in an increase in AR expression in both Ishikawa cells and HESCs as determined by western blot analysis (Fig. 4). In Ishikawa cells, metformin treatment reduced the testosterone-induced AR expression in a dose-dependent manner (Fig. 4, left panel). Similarly, in HESC cells, metformin treatment decreased the testosterone-induced AR expression; however, the effect was not dose-dependent (Fig. 4, right panel). Moreover, we examined the effect of metformin on AR expression under the same conditions using fluorescent immunocytochemistry. Testosterone treatment increased the fluorescence intensity of AR in both Ishikawa cells and HESCs (Fig. 5). Whereas, metformin treatment lowered the testosterone-induced fluorescence intensity in both the cell lines (Fig. 5).

### Metformin partly restores testosterone-reduced HOXA10 expression in the stromal cell line

**Fig. 6:**
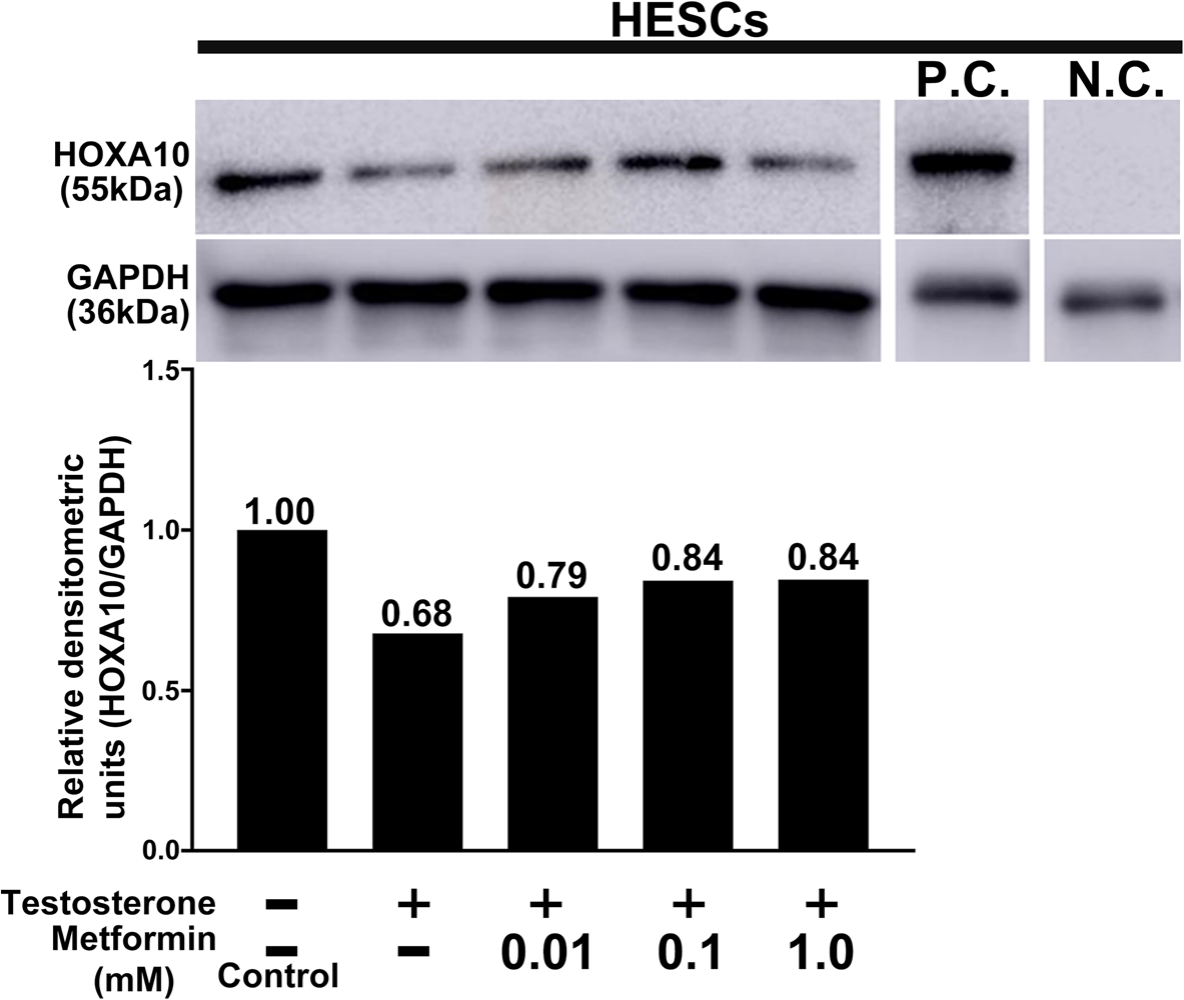
Effect of metformin treatment on HOXA10 expression of HESCs examined by western blotting. In the upper panel, the representative images show HOXA10 expression determined by western blotting in HESCs. Human Embryonic Kidney cells 293 whole cell lysate was used as a positive control, and samples without HOXA10 primary antibody were used as a negative control. Graphs in the lower panel show the relative densitometric units (HOXA10/GAPDH). The value in each column is the relative ratio when the control (without both testosterone and metformin) was set at 1.0. Three independent experiments showed the same results. HOXA10, homeobox A10; HESCs, human endometrial stromal cells; N.C., negative control; P.C., positive control

We investigated the direct effects of metformin on HOXA10 expression in endometrium in vitro. Testosterone treatment resulted in a decrease in HOXA10 expression in HESCs (Fig. 6). Meanwhile, metformin treatment only partially restored the testosterone-reduced HOXA10 expression in HESCs (Fig. 6).

## Discussion

In this study, we showed that metformin treatment resulted in a decrease in AR expression and an increase in HOXA10 expression in the endometrium of women with PCOS. Furthermore, we showed that while metformin counteracted the testosterone-induced AR expression in both Ishikawa cells and HESCs, metformin partly restored the testosterone-reduced HOXA10 expression in HESCs in vitro.

Aberrant expression of AR and HOXA10 in the endometrium may inhibit embryo implantation in women with PCOS. In non-PCOS women with normal ovulatory cycles, AR is expressed in the epithelium and stroma of the endometrium, and its expression decreases during the secretory phase [26]. Further, AR expression in the endometrium of women with PCOS is known to remain higher than that in non-PCOS women [26]. Moreover, our previous study has revealed that AR expression in the endometrium is high in women with PCOS [10]. In contrast, HOXA10, which is expressed in the stromal endometrium during the peri-implantation period, is indispensable for embryo implantation [12, 13]. Although the expression of HOXA10 in the endometrium is induced by ovarian steroid hormones [12, 13], testosterone reduces its expression in the endometrium. In vitro experiments using Ishikawa cells have revealed that HOXA10 mRNA expression decreases in a testosterone-dependent manner [16]. In addition, testosterone inhibits the mRNA expression of HOXA10 induced by estrogen, progesterone, or both [16]. Moreover, testosterone suppresses HOXA10 expression through an AR-mediated mechanism and its function is inhibited by flutamide, an AR antagonist [16]. Women with PCOS may be associated with hyperandrogenism, which may consequently induce aberrant expression of both AR and HOXA10 in the endometrium [12, 16]. In the present study, in vivo expressions of AR and HOXA10 in the endometrial tissues obtained from women with PCOS were consistent with those in previous reports [10, 16]. Thus, our present and previous results suggest that hyperandrogenemia may contribute to the reduced clinical pregnancy rate following embryo transfer as part of in vitro fertilization programs in women with PCOS by modulating expressions of AR and HOXA10.

The mechanism by which metformin, an insulin-sensitizing drug, reduces the testosterone level in women with PCOS may involve improvement in peripheral insulin resistance [27]. Metformin exerts its pharmacological effects mainly on the liver and skeletal muscles [28]. In women with PCOS, metformin reduces the peripheral insulin resistance, and the decreased insulin level improves hyperandrogenism by upregulating steroid hormone-binding proteins in the liver [27, 29]. In this study, metformin treatment decreased HOMA-IR and testosterone level in women with PCOS. These results are consistent with those in previous reports [10, 27, 29]. Decreased testosterone levels through reduced insulin level may have reduced the expression of AR in the endometrium of women with PCOS.

On the other hand, metformin acts directly on the endometrium to decrease AR expression. Wang et al. reported that metformin inhibits cell viability and apoptosis by targeting the AR signaling pathway [30]. The researchers used two prostate cancer cell lines, LNCaP and CWR22Rv1 cells, to prove that metformin does not affect the degradation or stability of AR protein; however, it suppresses the AR signaling pathway by repressing AR mRNA expression [30]. In contrast, metformin enters cells through membrane proteins called organic cation transporters (OCTs) to exert its effects. OCTs are reportedly closely associated with androgen signaling in many cells [31–33]. Although few studies have been conducted on the direct action of metformin on the endometrium in vivo so far, we showed that metformin treatment reduced testosterone-induced AR expression in the endometrium. These results, including ours, suggest that metformin has a direct effect on the endometrium and is involved in reducing AR expression in the endometrium of women with PCOS.

Additionally, the mechanism by which metformin upregulates the expression of HOXA10 in the endometrium of women with PCOS may be mediated through improvement in peripheral insulin resistance. As HOXA10 is suppressed by testosterone [16], it is possible that in this study, the decrease in testosterone levels improved HOXA10 expression in the endometrium. In addition, the endometrium samples collected after metformin administration in case 2 were from the secretory phase, thereby suggesting that the luteinizing effect of the ovulatory cycle recovery may have induced HOXA10 expression. In contrast, in vitro studies have shown that metformin partially restores HOXA10 expression in the presence of testosterone. This suggests that metformin may have a direct effect on HOXA10 expression in the endometrium.

However, there are no established mechanisms that explain how metformin directly affects the endometrium to regulate HOXA10 expression. Zhai et al. has reported that miR-491-3p and miR-1910-3p are potential microRNAs that regulate HOXA10 expression using differential microRNA and target scanning databases [34]. These researchers have reported that metformin may improve endometrial receptivity by downregulating the expression of miR-491-3p and miR-1910-3p and increasing the expression of HOXA10 in PCOS endometrium [34]. This suggests that metformin may have a direct effect on the endometrium.

Metformin treatment in Ishikawa cell reduced the testosterone-induced AR expression in a dose-dependent manner, however, the effect was not dose-dependent in HSECs. A recent study in mouse indicated that androgens increase endometrial gland formation and epithelial cell proliferation in both glandular and luminal compartments of the endometrium [35]. Moreover, a study investigating the effects of dihydrotestosterone demonstrated that epithelial and stromal cells play different roles in endometrial function [36]. These reports suggest that the responsiveness to androgens or ARs may differ between endometrial epithelial cells and stromal cells. Although the effect of metformin on AR signaling pathway remains to be fully clarified, cell-specificity might be present in Ishikawa cells and HSECs.

The present study has certain limitations. First, the sample size in this study was limited because it was difficult to get consent for endometrial sampling from patients with PCOS. Since PCOS is a heterogeneous condition marked by reproductive, endocrine, and metabolic abnormalities, it should be noted that our results may not be representative of all PCOS cases. In addition, this study did not take menstrual cycle into consideration while following the experimental protocol. The patient in case 2 changed from proliferative to secretory phase while collecting the sample after metformin treatment. Western blotting has revealed a lower endometrial expression of AR in the secretory phase than that in the proliferative phase in PCOS patients [37]. Therefore, although we observed a decrease in AR expression in case 2 after metformin treatment, it is possible that the change was caused by the menstrual cycle. Our study has limited to the small sample size for different menstrual cycle. Further studies are needed for validating the effects of metformin on endometrial AR expression with PCOS, with more cases focusing on the menstrual cycle. Second, we used cell lines under testosterone administration as in vitro models of the endometrial environment in PCOS. However, whether this is a valid in vitro model remains to be examined, and further in vivo studies using primary cell cultures from PCOS patients are needed. Third, in addition to HOXA10, many other important molecules such as leukemia inhibitory factor, epidermal growth factor, colony stimulating factor-1, and interleukin-1 are expressed in the endometrial epithelial and stromal cells during implantation. Thus, it is necessary to explore how metformin acts on the expression of these molecules as well.

## Conclusions

We showed that metformin treatment resulted in a decrease in AR expression and an increase in HOXA10 expression in the endometrium of women with PCOS. Moreover, we showed that metformin counteracted testosterone-induced AR expression in both Ishikawa cells and HESCs; whereas, metformin partly restored the testosterone-reduced HOXA10 expression in HSECs in vitro. Metformin may exert not only indirect but also direct effects on the endometrium, as a result, it may ameliorate the abnormal expression of AR and HOXA10 in the endometrium of patients with PCOS. However, further extensive research on the effects of metformin on the endometrium of patients with PCOS is needed to clarify the cause of endometrial abnormalities.

## Data Availability

All data generated or analyzed during this study are included in this published article.

## Abbreviations

AR: Androgen receptor
DAPI: 4′,6-diamidino-2-phenylindole
DMEM: Dulbecco’s modified Eagle’s medium
FBS: Fetal bovine serum
HESCs: Human endometrial stromal cells
HOMA-IR: Homeostasis model assessment-insulin resistance
HOXA10: Homeobox A10
HSCORE: Histochemical scoring system
OCT: Organic cation transporter
PBS: Phosphate-buffered saline
PCOS: Polycystic ovary syndrome
PVDF: Polyvinylidene difluoride
